# Postactivation Potentiation Biases Maximal Isometric Strength Assessment

**DOI:** 10.1155/2014/126961

**Published:** 2014-07-15

**Authors:** Leonardo Coelho Rabello Lima, Felipe Bruno Dias Oliveira, Thiago Pires Oliveira, Claudio de Oliveira Assumpção, Camila Coelho Greco, Adalgiso Croscato Cardozo, Benedito Sérgio Denadai

**Affiliations:** ^1^Human Performance Laboratory, Physical Education Department, São Paulo State University, Rio Claro, SP, Brazil; ^2^Biomechanics Laboratory, Physical Education Department, São Paulo State University, Rio Claro, SP, Brazil

## Abstract

Postactivation potentiation (PAP) is known to enhance force production. Maximal isometric strength assessment protocols usually consist of two or more maximal voluntary isometric contractions (MVCs). The objective of this study was to determine if PAP would influence isometric strength assessment. Healthy male volunteers (*n* = 23) performed two five-second MVCs separated by a 180-seconds interval. Changes in isometric peak torque (IPT), time to achieve it (tPTI), contractile impulse (CI), root mean square of the electromyographic signal during PTI (RMS), and rate of torque development (RTD), in different intervals, were measured. Significant increases in IPT (240.6 ± 55.7 N*·*m *versus* 248.9 ± 55.1 N*·*m), RTD (746 ± 152 N*·*m*·*s^−1^ *versus* 727 ± 158 N*·*m*·*s^−1^), and RMS (59.1 ± 12.2% RMS_MAX_ *versus* 54.8 ± 9.4% RMS_MAX_) were found on the second MVC. tPTI decreased significantly on the second MVC (2373 ± 1200 ms *versus* 2784 ± 1226 ms). We conclude that a first MVC leads to PAP that elicits significant enhancements in strength-related variables of a second MVC performed 180 seconds later. If disconsidered, this phenomenon might bias maximal isometric strength assessment, overestimating some of these variables.

## 1. Introduction

Postactivation potentiation (PAP) is a widely studied phenomenon that occurs when the neuromuscular system gets in a potentiated state after an applied stimulus [1]. The types of stimuli vary from isometric [2–4] and dynamic contractions [5–7] to whole-body vibration [8, 9] and electrical stimulation [10] (i.e., posttetanic potentiation), which can lead to augmented power output in further activities like explosive contractions and sport-specific actions [1, 11, 12]. Some authors also found increases in maximal force production [13], even though most of the studies in the area present evidence for potentiated muscular power.

There are three major theories that explain the underlying physiological mechanisms of PAP [1]. One of the most consistent theories states that prior stimulation phosphorylates the myosin's regulatory light chains, moving them away from the myosin thick body and closer to the actin filaments [14], also increasing its sensitivity to Ca^2+^, which facilitate interactions within the sarcomeric apparatus [15]. Another consistent theory considers that preconditioning activities are responsible for increasing transmittance of excitation potentials in the synaptic junction and spinal cord levels [16–18]. In order to try to better understand this phenomenon, a considerable number of studies assess electromyographic data in potentiated muscles [2, 16, 18, 19]. A third theory suggests that a reduction in the pennation angle, induced by a potentiating stimulus, may contribute to increases in power and strength, once this change permits a more direct transmission from the muscle-fiber forces to the tendon. However, to our knowledge, there is only one study presenting solid evidence of this phenomenon [20].

In order to obtain optimal PAP response, some stimulation variables should be considered. There is an optimal interval between stimulation and assessment, which varies depending on the intensity and type of the stimulus [21–23]. This interval must be long enough to promote recovery from acute metabolic fatigue and short enough to assess the muscle in a potentiated state (that disappears approximately 15 minutes after stimulation) [1, 24]. In most studies, the interval between stimulation and assessment ranges between 3 and 8 minutes [2–6]. It is also known that neuromuscular factors such as strength levels, fiber type distribution, training level, and power/strength ratio play important roles on an individual's responsiveness to PAP [1, 25].

Strength production is one of the most important criterion measures adopted in studies that investigate the neuromuscular system [26–28]. A considerable number of studies use maximal isometric voluntary contraction (MVC) to assess muscular strength due to the number of variables that can be obtained from it (i.e., isometric peak torque (IPT), contractile impulse (CI), rate of torque development (RTD), etc.) [2, 29, 30]. For instance, RTD can be assessed in different time epochs from contraction onset (i.e., 10 to 250 ms), providing information regarding the nature of the adaptation in explosive force. Indeed, the RTD assessed at early phase (<100 ms) is influenced by intrinsic muscle contractile properties and neural drive, whereas the late phase (>100 ms) is influenced by muscle cross-sectional area, neural drive, and stiffness of tendon-aponeurosis complex [29]. Many strength assessment protocols adopt a predetermined number of MVCs separated by fixed rest intervals, using the contraction with the greatest isometric peak torque for analysis [29–32]. To the best of our knowledge, there are no studies reporting on which of the contractions is the most frequently analyzed. In that way, many of the isometric strength assessment protocols resemble potentiating protocols, using 3–5-second contractions separated by 30–60 seconds [29–34].

Considering the similarity between PAP stimulation and strength assessment protocols, our goal was to investigate whether PAP would bias isometric strength assessment (i.e., MVC), using a protocol involving two MVCs separated by a 180-second rest interval. We hypothesized that a first MVC would potentiate the neuromuscular system, altering strength-related markers of a second MVC and, most importantly, influencing the selection of the analyzed contraction.

## 2. Materials and Methods

### 2.1. Subjects

Twenty-three males (23.5 ± 4 yr, 177 ± 8 cm, 77.2 ± 13.4 kg, and 24.8 ± 4.1 kg/m^2^) volunteered for the study. One of the criteria for participation in the study was not to be engaged in any regular physical activity for the last 6 months, as well as not having any recent injury in the knee joint. The volunteers were asked not to take part in any physical activities other than those required on their daily routines during the study. All volunteers were informed about their rights as research subjects as well as the inherent risks of all the procedures by reading and signing an informed term of consent approved by the University Institutional Review Board for Human Subjects.

### 2.2. Experimental Design

The volunteers visited the laboratory on three occasions, each separated by 3–5 days. Two of these visits were used as familiarization sessions to lessen any effect of learning during subsequent strength testing. During these sessions, volunteers performed a nonspecific (8 min running at 8 km/h) and a specific warm-up (3 submaximal isometric contractions) before performing one MVC for the knee extensors in the dynamometer (Biodex System 3, Biodex Medical Systems, Shirley, NY). The third visit consisted of the experimental session, in which the volunteers followed the same warm-up procedure and, then, performed two MVCs for knee extensors in the isokinetic dynamometer to determine all criterion measures (IPT, RTD, RMS, and CI). T Seat set-up was recorded in the first familiarization session and reproduced in the following sessions. Volunteers were instructed to arrive at the laboratory in a rested and fully hydrated state, at least 3 h postprandially. They were also asked not to do any strenuous activity during the day prior to each test. All tests were performed in a climate-controlled (21-22°C) laboratory at the same time of the day (±2 h) to minimize the effects of diurnal biological variation on the results [35].

### 2.3. Familiarization Sessions

During the two familiarization trials, all volunteers were fully instructed and habituated to the test procedures in the dynamometer in order to avoid strength underestimation [36]. Prior to testing, they performed a standardized warm-up period of 8 min running at 8 km/h followed by 3 submaximal isometric contractions of the knee extensors. Subsequently, each subject was carefully instructed to contract “as fast and forcefully as possible” when performing one five-second MVC.

### 2.4. Determination of the MVCs

The dynamometer and its specific computer software were used to measure MVC. Volunteers were placed in a sitting position and securely strapped into the test chair. Extraneous movement of the upper body was limited by two crossover shoulder harnesses and an abdominal belt. The trunk/thigh angle was 85°. The axis of the dynamometer was lined up with the right knee flexion-extension axis, and the lever arm was attached to the shank by a strap. The volunteers were asked to relax their legs so that passive determination of the effects of gravity on the limb and lever arm could be carried out. MVCs were performed for the knee extensors (m. quadriceps femoris) at a static knee joint angle of 75° (0° = full extension) [29, 30]. Two maximal isometric attempts were performed with rest periods of 180 s in between, which would allow proper recovery of the intracellular phosphocreatine reserves. The volunteers were instructed to extend their knees “as fast and forcefully as possible” [29] and each MVC was sustained for 5 s.

### 2.5. Surface Electromyographic Recordings

The electromyographic (EMG) signal from the vastus lateralis muscle of the assessed limb was collected during all MVCs performed in the experimental session via disposable adherent Ag/AgCl bipolar electrodes (20 mm between poles) placed over the skin surface of the muscle and one simple reference electrode placed over the ulnar styloid process, both were connected to a preamplifier (100 times gain), following the SENIAM recommendations for electrode placement and EMG assessment [37], which were connected to a signal acquisition module (EMG System, Brazil) (20 times gain). Prior to electrode application, the skin was shaved, abraded, and cleansed with alcohol. The gain promoted by both the data acquisition system and the preamplifier enhanced the original biologic value 2000 times. Specific software was used to assess the EMG data with a 1000 Hz frequency.

### 2.6. Data Processing

The torque data of the contractions was obtained at a 1000 Hz frequency by a biological signal acquisition module (EMG System) synchronized with the isokinetic dynamometer. The data was filtered (Butterworth filter, low pass, 4th order, with a 15 Hz cut-off frequency) and analyzed in the MatLab 6.5 software (Mathworks, USA). The isometric peak torque (IPT) was considered the highest value in the torque-time curve, and the time point when it happened was also considered for analysis (tIPT). Contractile impulse (CI) was also calculated as the area under the torque-time curve, using trapezoidal integration (∫_*a*_^*b*^ *f*(*x*)*dx*). [29]. Rate of torque development (RTD) was obtained by the slope of the isometric contraction torque-time curve (i.e., Δtorque*·*Δtime^−1^) across the time intervals of 0–30, 0–50, 0–100, 0–150, 0–200, and 0–250 ms relative to the onset of contraction. Maximal rate of torque development (RTD_MAX_) was also calculated as the steepest point of the torque-time curve. The onset of muscle contraction was defined as the time point where the knee extensors torque exceeded the baseline by 2.5% of the baseline-to-peak difference [31].

The EMG data analyses were performed in the MatLab 6.5 software in which a high pass (Butterworth, high pass, 2nd order, with a 20 Hz cut-off frequency) and a low pass (Butterworth, low pass, 4th order, with a 500 Hz cut-off frequency) filters were applied. The average root mean square (RMS) value of the EMG signal obtained at a 0.5-second interval during the MVC (i.e., 0.25 seconds before and 0.25 seconds after the IPT) was normalized by the peak RMS value obtained at the same time interval during EMG signal of the first experimental MVC. In that manner, all EMG values were expressed as percentages of the maximal RMS value (%RMS_MAX_) of the first experimental contraction.

### 2.7. Statistical Analyses

All data are expressed as means ± standard deviation (SD). Data normality was tested with the Shapiro-Wilk test. A one-way repeated-measures ANOVA was used to compare IPT obtained during familiarization sessions and at the first trial of the experimental session in order to identify possible familiarization and learning effects. Differences in all criterion measures (IPT, tIPT, CI, RTD, and EMG) obtained during the experimental session were identified using Student's *t*-tests for paired samples. Relationships between changes (2nd MVC and 1st MVC) in key dependent variables were assessed using Pearson's product moment correlation coefficients. The significance level was set at *P* ≤ 0.05.

## 3. Results

**Figure 1:**
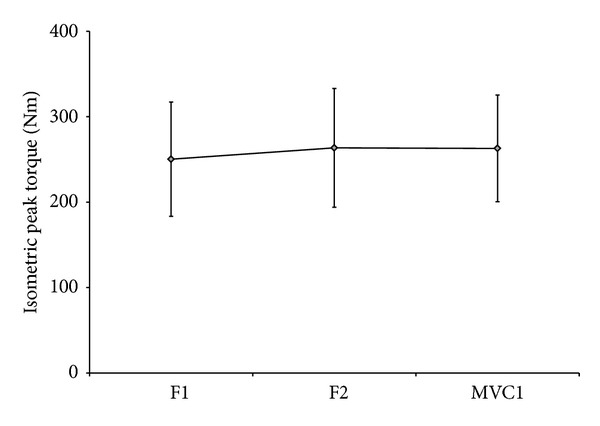
Mean (SD) isometric peak torque values obtained at the two familiarization sessions and the first maximal voluntary contraction. F1: first familiarization session; F2: second familiarization session; MVC1: first experimental maximal voluntary isometric contraction.

The IPT values for both familiarization sessions were not significantly different than the IPT obtained at the first experimental session (Figure 1), which means that learning and adaptation to the tests did not bias the strength assessment.

**Figure 2:**
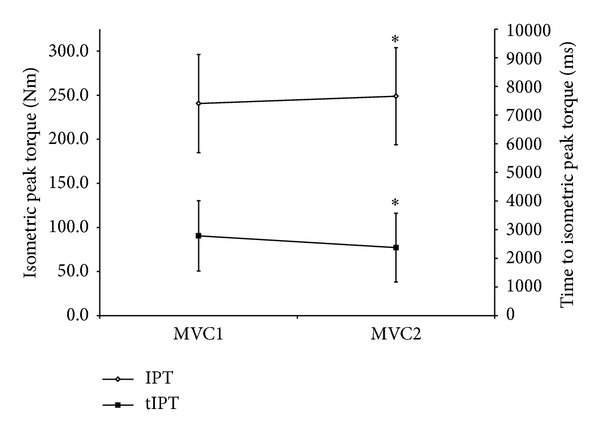
Mean (SD) isometric peak torque and time to reach peak torque values of the first and second maximal voluntary isometric contractions. MVC1: first maximal voluntary isometric contraction; MVC2: second maximal voluntary isometric contraction; ^∗^significantly different from the first contraction.

IPT and tIPT values are expressed in Figure 2. IPT values of the second trial were significantly (*P* = 0.0003) higher than those obtained at the first trial (240.6 ± 55.7 N*·*m* versus *248.9 ± 55.1 N*·*m). Also, the tPTI of the second trial (2373 ± 1200 ms) was significantly (*P* = 0.02) lower than the tPTI of the first trial (2784 ± 1226 ms). No significant alterations (*P* > 0.05) were found for CI (1st trial: 1086 ± 274 N*·*m* versus* 2nd trial: 1106 ± 286 N*·*m).

**Figure 3:**
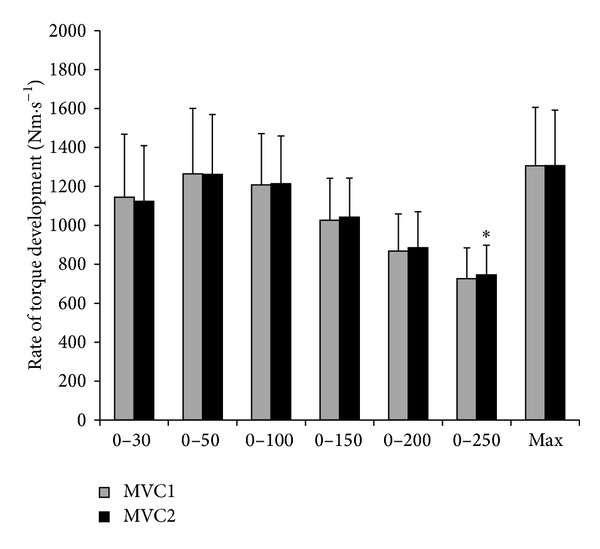
Mean (SD) rate of torque development in different intervals of the first and second maximal voluntary isometric contractions. MVC1: first maximal voluntary isometric contraction; MVC2: second maximal voluntary isometric contraction; ^∗^significantly different from the first contraction.

There was a significant (*P* = 0.04) increase in the normalized RMS of the EMG signal of the vastus lateralis muscle during the second trial (59.1 ± 12.2% RMS_MAX_) when compared to the first trial (54.8 ± 9.4% RMS_MAX_). RTD_MAX_ did not present significant increases (1st trial: 1306 ± 308 N*·*m*·*s^−1^ *versus* 2nd trial: 1307 ± 292 N*·*m*·*s^−1^). However, RTD_250_ was significantly (*P* = 0.04) higher in the second trial (746 ± 152 N*·*m*·*s^−1^) when compared to the first trial (727 ± 158 N*·*m*·*s^−1^). RTD analyzed in all other intervals was not significantly increased at the second MVC (Figure 3). Additionally, a significant correlation (*r* = 0.54; *P* = 0.01) was found for increases in IPT and EMG signal.

## 4. Discussion

The aim of the present study was to investigate if PAP would bias isometric strength assessment performed by a protocol consisting of two MVCs separated by a 180-second rest interval. No learning effect was identified during the study, since IPT values for both familiarization sessions were not significantly different than those obtained at the first experimental MVC. Significant differences in IPT, tIPT, RMS, and RTD (relative to the 0–250 ms interval) were found between the two experimental MVCs, showing that PAP elicited increases/decreases in strength-related criterion measures.

It has been well established that familiarization sessions are an important methodological concern amongst studies that investigate isometric [38] and dynamic [39–41] muscular strength. Indeed, studies that investigate PAP [8, 42, 43] adopt familiarization sessions before the protocol in order to avoid over- and underestimation in strength assessment [39]. Although most studies use single familiarization before engaging in experimental sessions and applying potentiating stimuli [8, 42], we chose to add an extra session to the familiarization protocol, considering the data from Wallerstein et al. [38], which showed stability in IPT values only after a third familiarization session in the elderly (i.e., the IPT of the fourth session was not different from the third). However, adopting IPT as criteria for stabilization, we found that either one or two familiarization sessions were enough to attend to this purpose in young adults. To our knowledge, there are no studies that investigated how many familiarization sessions are necessary to reach IPT stability amongst young males. Nevertheless, reproducibility and validity of strength assessment in isokinetic dynamometers have been otherwise described [44, 45].

PAP is known to significantly enhance muscular power assessed through isokinetic contractions [2], jumps [2, 3, 5], and other athletic activities [11]. However, in the present study, a previous MVC led to 3.5% increases in IPT of a second MVC performed 180 seconds later. Few are the studies that investigated the influence of PAP on isometric contractions [42, 46, 47]. Fukutani et al. [46] and Miyamoto et al. [47], for instance, found increased twitch isometric torque values after isokinetic contractions. Both of these studies showed that PAP induced by dynamic activities can enhance maximal force production. This enhancement in maximal isometric strength can be partially explained by changes in the pennation angle, one of the proposed mechanisms for PAP [20]. Moreover, a 4,5% increase in the activation of the vastus lateralis was also identified during this study, which could have happened due to increased transmittance in the synaptic junctions of the motoneuron [16, 17] or decreased synaptic failures, leading to the recruitment of a greater number of motor units [18]. Indeed, a significant moderate correlation was found between the increases in IPT and RMS of the vastus lateralis, contributing to this rationale. However, the common variance (*r* ^2^ = 0.29) was low, which could not provide a cause-effect relationship between the increases of IPT and RMS.

Although IPT increased significantly in the second MVC, CI values were not significantly different between each other, which means that PAP might elicit increases in maximal force production but not in its maintenance whatsoever. However, adding to the knowledge that PAP increases explosive muscular actions [2–7], the tPTI decreased significantly during the second MVC. This alteration might be explained by the phosphorylation of the myosin light chain, rendering structural changes within the sarcomeres and augmented sensitivity to Ca^2+^, as reported by Szczesna et al. [14].

Besides tPTI, the main variable adopted in this study to investigate explosive force was RTD (maximal and on fixed intervals). Although IPT increased after the first MVC, maximal RTD was not influenced by the PAP elicited by a prior contraction. Other authors identify a potentiating effect caused by dynamic contractions on the RTD [42, 43]. For instance, Gilbert and Lees [42] found that RTD measured by MVC is potentiated after both maximal strength and power exercises. They also showed that a longer period (~15–20 minutes) than that adopted in the present study might be necessary for the potentiation of RTD after maximal strength exercise (1RM squat). Baudry and Duchateau [43] also found increased maximal RTD after a six-second ballistic MVC of the thumb adductor showing, however, that maximal RTD measured by twitch contractions might be greatly influenced by PAP, since it does not depend on metabolic factors such as complete restoration of the intracellular phosphocreatine reserves within the muscle. On the other hand, Bazett-Jones et al. [48] found that maximal RTD was not enhanced one minute after a potentiating protocol consisting of three dynamic contractions with a load of 90% 1RM. Instead, RTD decreased significantly after the conditioning protocol.

To the best of our knowledge, there are no studies investigating the effects of PAP on the RTD measured in classically adopted fixed intervals (0–30, 0–50, 0–100, 0–200, and 0–250 ms) [29, 30]. The results obtained in our study showed that, of all analyzed intervals, the only one to respond to PAP was 0–250 ms. Although all RTD measurements represent explosive strength, its manifestation in early intervals (i.e., 0–30 to 0–100 ms) seems to be related to neural factors such as motoneuron recruitment, firing frequencies, and Ca^2+^ kinetics, while latter intervals seem to be more related to maximal strength production [29]. In that way, the increases in RTD in the 0–250 ms interval are coherent with the increases in IPT and EMG observed in our study.

Validity and reliability are paramount in strength assessment [49, 50]. One of the biggest concerns in investigations that involve sensible strength variables such as RTD is to ensure proper placement and fixation of the subjects on the strength measuring device [29], as well as the maintenance of the exact same testing conditions. With that assured, many researchers adopt the MVC with the greatest IPT value for analyses [29–32], disregarding potential PAP and/or fatigue effects during the protocol. Our data showed that, using a protocol consisting of two MVCs separated by a 180-second rest interval, PAP might occur increasing IPT values and biasing the selection of the analyzed MVC. Moreover, in investigations that consider explosive strength and EMG signal, the potentiating effects of a first MVC might also influence important variables like RTD measured in different time intervals and RMS values. Notwithstanding, there are other factors that might affect PAP. It is known that fiber type distribution, strength levels, and power/strength ratio are important factors that contribute to an individuals' responsiveness to PAP [1, 25]. Therefore, in study models that involve neuromuscular training, not only could PAP bias initial strength assessment, but also influence post-training measurements, eliciting higher increases in strength-related variables for groups that experienced training interventions. This increased PAP responsiveness could be responsible for magnifying the effects of training, as the control group would remain in the same training state, responding similarly to PAP.

## 5. Conclusion

We conclude that the strength assessment protocol we adopted in the present study might be biased by PAP, since a first MVC renders the neuromuscular system in a potentiated state, while the rest intervals allow enough time for the ATP-CP system to partially recover, resulting in an enhanced strength response. Moreover, it is important to note that study protocols involving strength training might also be biased by PAP, since well-trained individuals respond better to this phenomenon. Therefore, we suggest that researchers and practitioners consider potential PAP effects on isometric strength assessment protocols and take extra care in the analyses process to avoid confounding data.
